# Effect of aspirin treatment duration on clinical outcomes in acute coronary syndrome patients with early aspirin discontinuation and received P2Y12 inhibitor monotherapy

**DOI:** 10.1371/journal.pone.0251109

**Published:** 2021-05-12

**Authors:** Ming-Yun Ho, Po-Wei Chen, Wen-Han Feng, Chun-Hung Su, Sheng-Wei Huang, Chung-Wei Cheng, Hung-I Yeh, Ching-Pei Chen, Wei-Chun Huang, Ching-Chang Fang, Hui-Wen Lin, Sheng-Hsiang Lin, I-Chang Hsieh, Yi-Heng Li

**Affiliations:** 1 Chang Gung Memorial Hospital, Chang Gung University College of Medicine, Taoyuan, Taiwan; 2 National Cheng Kung University Hospital, College of Medicine, National Cheng Kung University, Tainan, Taiwan; 3 Kaohsiung Municipal Ta-Tung Hospital and Kaohsiung Medical University Hospital, Kaohsiung, Taiwan; 4 Chung Shan Medical University Hospital and Chung Shan Medical University, Taichung, Taiwan; 5 MacKay Memorial Hospital, Taipei, Taiwan; 6 Changhua Christian Hospital, Changhua, Taiwan; 7 Kaohsiung Veterans General Hospital, Fooyin University, Kaohsiung and National Yang Ming University, Taipei, Taiwan; 8 Tainan Municipal Hospital, Tainan, Taiwan; 9 Institute of Clinical Medicine, College of Medicine, National Cheng Kung University, Tainan, Taiwan; 10 Department of Public Health, College of Medicine, National Cheng Kung University, Tainan, Taiwan; 11 Biostatistics Consulting Center, National Cheng Kung University Hospital, College of Medicine, National Cheng Kung University, Tainan, Taiwan; Federico II University, ITALY

## Abstract

Recent clinical trials showed that short aspirin duration (1 or 3 months) in dual antiplatelet therapy (DAPT) followed by P2Y12 inhibitor monotherapy reduced the risk of bleeding and did not increase the ischemic risk compared to 12-month DAPT in acute coronary syndrome (ACS) patients undergoing percutaneous coronary intervention (PCI). However, it is unclear about the optimal duration of aspirin in P2Y12 inhibitor monotherapy. The purpose of this study was to evaluate the influence of aspirin treatment duration on clinical outcomes in a cohort of ACS patients with early aspirin interruption and received P2Y12 inhibitor monotherapy. From January 1, 2014 to December 31, 2018, we included 498 ACS patients (age 70.18 ± 12.84 years, 71.3% men) with aspirin stopped for various reasons before 6 months after PCI and received P2Y12 inhibitor monotherapy. The clinical outcomes between those with aspirin treatment ≤ 1 month and > 1 month were compared in 12-month follow up after PCI. Inverse probability of treatment weighting was used to balance the covariates between groups. The mean duration of aspirin treatment was 7.52 ± 8.10 days vs. 98.05 ± 56.70 days in the 2 groups (p<0.001). The primary composite endpoint of all-cause mortality, recurrent ACS or unplanned revascularization and stroke occurred in 12.6% and 14.4% in the 2 groups (adjusted HR 1.19, 95% CI 0.85–1.68). The safety outcome of BARC 3 or 5 bleeding was also similar (adjusted HR 0.69, 95% CI 0.34–1.40) between the 2 groups. In conclusion, patients with ≤ 1 month aspirin treatment had similar clinical outcomes to those with treatment > 1 month. Our results indicated that ≤ 1-month aspirin may be enough in P2Y12 inhibitor monotherapy strategy for ACS patients undergoing PCI.

## Introduction

Current guidelines recommend 12-month dual antiplatelet therapy (DAPT) with aspirin and a P2Y12 inhibitor for patients with acute coronary syndromes (ACS) undergoing percutaneous coronary intervention (PCI) [1, 2]. However, the optimal duration of DAPT is still controversial because the DAPT-associated bleeding is a major clinical challenge. Multiple lines of evidence have shown that major bleeding is a significant risk factor for cardiac morbidity and mortality in patients with ACS or after coronary stenting [3, 4]. The platelet inhibitory effects are greater with P2Y12 inhibitors than aspirin. Aspirin provides little additional antiplatelet effects under P2Y12 inhibitor treatment [5, 6]. Therefore, one of the ways to decrease bleeding risk while preserving antithrombotic efficacy is to abandon aspirin early after PCI and use P2Y12 inhibitor monotherapy [5, 6]. Recently, this strategy of short-term DAPT (aspirin 1 or 3 months) followed by P2Y12 inhibitor monotherapy was evaluated in a number of clinical trials. These studies demonstrated that P2Y12 inhibitor monotherapy could be an effective and safe antiplatelet strategy in patients undergoing PCI [7–11]. A meta-analysis, involving these 5 clinical trials (GLOBAL-LEADERS, TWILIGHT, SMART-CHOICE, STOPDAPT-2, and TICO) with 32,361 patients, provided strong evidence that P2Y12 inhibitor monotherapy results in significantly lower rate of bleeding compared with conventional 12-month DAPT with no signal of increased ischemic risk [12]. In the TWILIGHT, SMART-CHOICE and TICO trials, the DAPT duration was 3 months; while the length of DAPT was only 1 month in GLOBAL-LEADERS and STOPDAPT-2 trials. When P2Y12 inhibitor monotherapy is considered as an alternative antiplatelet strategy for ACS patients at bleeding risk, it is still unclear about the optimal duration of aspirin. We designed this study to include ACS patients who underwent PCI but only received short duration of aspirin for various reasons and received P2Y12 inhibitor monotherapy. The clinical outcomes were compared between those with aspirin treatment duration ≤ 1 month and > 1 month after PCI and switching to P2Y12 inhibitor monotherapy.

## Methods

### Study population

The design of this multicenter, retrospective, observational study was published previously [13]. In brief, ACS patients who received PCI during admission and were treated with P2Y12 inhibitor monotherapy were enrolled from January 2014 to December 2018 from 8 major teaching hospitals in Taiwan. Patients were eligible if they were ≥ 18 years, admitted with a major diagnosis of ACS, received PCI with bare metal stent (BMS) and/or contemporary drug eluting stent (DES) implantation during hospitalization, survived to discharge, and regularly followed up in outpatient clinic for at least 1 year after discharge. A patient could receive more than one stent during PCI. Aspirin was stopped within 6 months after PCI in all included patients due to various reasons. P2Y12 inhibitor monotherapy with either clopidogrel 75 mg daily or ticagrelor 90 mg twice daily was used. The exclusion criteria were patients with (1) life-threatening malignancy with life expectancy less than 1 year, (2) hematological disease with bleeding tendency, (3) treatment with immunosuppressive agents, and (4) need of oral anticoagulation therapy. The demographic data, coronary risk factors, major disease history, PCI procedures and medications were collected from the patients’ medical records according to a pre-determined study protocol. The timing and reasons for aspirin discontinuation after PCI were recorded. Enrolled patients were divided into 2 groups by the timing of aspirin withdrawal: ≤ 1 month or > 1 month after PCI. The study was conducted according to the principles expressed in the Declaration of Helsinki and was approved by the Institutional Review Boards of the 8 participating hospitals. All data from the medical records were fully anonymized. The study protocol was approved by the Medical Ethics Committee of National Cheng Kung University Hospital (IRB: A-ER-107-375) and granted a waiver of informed consent due to its study nature.

### Follow-up

All patients were followed up for at least 12 months after discharge or until one of the primary composite endpoints occurred. The primary composite endpoints included all-cause mortality, recurrent ACS or unplanned revascularization, and stroke within 12 months after the index PCI. The secondary endpoint was the breakdown incidence of the primary composite endpoints. Recurrent ACS was defined as readmission to a hospital for a primary diagnosis of new onset ST-segment elevation myocardial infarction (STEMI), non-ST segment elevation myocardial infarction (NSTEMI) or unstable angina. Unplanned revascularization was defined as the first unexpected revascularization after discharge, including redo PCI or coronary artery bypass graft (CABG) after the index PCI due to new onset ischemic symptoms. Stroke, including ischemic or hemorrhagic stroke, was diagnosed by the occurrence of new-onset neurological symptoms and signs with neuroimaging studies. All clinical events of the primary composite endpoints were documented in the medical records and reported by the physicians that followed up the patients. The safety endpoint was the occurrence of major bleeding, which was defined as the Bleeding Academic Research Consortium (BARC) type 3 and 5 bleedings [14].

### Statistical analysis

Continuous variables were expressed as mean ± standard deviation and categorical variables were expressed as numbers and percentages. Unpaired Student’s t test for continuous variables and chi-square test for categorical variables were used for comparison between groups. The level of statistical significance was set at p < 0.05 (2-tailed). To adjust for potential confounding due to baseline imbalances in study covariates while preserving sample size, we used the inverse probability of treatment weights (IPTW) method based on the propensity score. The propensity score is the probability conditional on baseline covariates, including age, sex, STEMI status, diabetes mellitus, hypertension, hyperlipidemia, smoker, previous MI, previous PCI, previous CABG, previous ischemic stroke, previous hemorrhagic stroke, chronic kidney disease without dialysis, end stage renal disease with dialysis, heart failure, atrial fibrillation, peripheral artery disease, left ventricular ejection fraction, coronary angiography finding, PCI procedure, location of lesion treated, stent, and medications. With IPTW method, the propensity score was used to generate patient specific stabilized weights that control for covariate imbalances [15, 16]. The propensity-score weight was calculated as the inverse of the propensity score for each client. Both the absolute standardized mean difference (ASMD) and p value were used to assess the balance between groups before and after weighting. The Cox proportional-hazards models were then adjusted for differences in the treatment groups using IPTW derived from the propensity score which was designated as IPTW model. In the IPTW model after matching, the clinical factors with ASMD > 0.1 were put into the multivariate Cox proportional-hazards models for further adjustment. Adjusted hazard ratios (HRs) and 95% confidence intervals (CIs) were calculated. We used the same Cox proportional hazards model to estimate p values for interaction in the subgroup analysis. SAS statistical package (version 9.4 for Windows; SAS Institute, Cary, NC, USA) was used for all analyses.

## Results

Overall, a total 498 patients (mean age 70.18 ± 12.84 years, men 71.3%) that fulfilled the inclusion and exclusion criteria were included in this study. The mean duration of aspirin treatment was 40.25 ± 55.63 days. There were 318 patients (63.9%) whose aspirin was stopped before 1 month after PCI. The mean time of aspirin treatment was 7.52 ± 8.10 days in those with aspirin treatment duration ≤ 1 month and 98.05 ± 56.70 days in those with > 1 month (p < 0.001). **Table 1**shows the comparisons of baseline characteristics of patients between the 2 groups. Among all patients, 28.3% had STEMI, 54.4% had diabetes, and 49.8% had chronic kidney disease, including 13.7% receiving dialysis. For PCI procedure, 44.2% were intervention of multiple lesions and 57% received DES. The percentage of P2Y12 inhibitors, including 54.4% clopidogrel and 45.6% ticagrelor, were similar between the 2 groups. After propensity score matching, the 2 groups were almost balanced in clinical characteristics and intervention procedures **(Table 1)**. **Table 2**illustrates the reasons for premature discontinuation of aspirin. The most common reason to stop aspirin was gastrointestinal bleeding (46.59%) with a similar percentage in both groups. Aspirin allergy or intolerance and gastrointestinal upset were also common reasons to stop aspirin. Aspirin allergy or intolerance was more common in those with aspirin treatment duration ≤ 1 month; while gastrointestinal upset and discomfort were higher in those with > 1 month. There were 23.49% patients that had other or unknown causes.

**Table 1:** Baseline characteristics of patients with different duration of aspirin use.

			Inverse probability of treatment weighting									
			Before						After			
	All		≤ 1 month		> 1 months		*p* value	ASMD	≤ 1 month	> 1 months	*p* value	ASMD
	N = 498	(%)	N = 318	(%)	N = 180	(%)			(pseudo data)	(pseudo data)		
Age	70.18 ± 12.84		71.00 ± 12.57		68.74 ± 13.20		0.059	0.175	70.24 ± 16.05	71.45 ± 22.22	0.319	0.063
Male	355	71.29	237	74.53	118	65.56	0.043	0.197	71.53	70.9	0.904	0.014
STEMI	141	28.31	89	27.99	52	28.89	0.912	0.020	28.21	23.99	0.364	0.096
Diabetes mellitus	271	54.42	173	54.40	98	54.44	1.000	0.001	54.80	59.78	0.400	0.101
Hypertension	376	75.50	233	73.27	143	79.44	0.153	0.146	75.27	76.39	0.821	0.026
Hyperlipidemia	273	54.82	170	53.46	103	57.22	0.473	0.076	54.36	51.75	0.683	0.052
Smoker	146	29.32	101	31.76	45	25.00	0.136	0.150	28.98	25.05	0.415	0.089
Previous MI	78	15.66	50	15.72	28	15.56	1.000	0.005	14.98	13.99	0.786	0.028
Previous PCI	140	28.11	103	32.39	37	20.56	0.007	0.271	27.00	32.78	0.424	0.127
Previous CABG	16	3.21	11	3.46	5	2.78	0.881	0.039	2.94	3.07	0.946	0.008
Previous ischemic stroke	76	15.26	49	15.41	27	15.00	1.000	0.011	16.22	14.99	0.753	0.034
Previous hemorrhagic stroke	3	0.60	2	0.63	1	0.56	1.000	0.010	0.57	0.46	0.857	0.015
CKD without dialysis	180	36.14	120	37.74	60	33.33	0.376	0.092	36.06	37.15	0.866	0.023
ESRD with dialysis	68	13.65	39	12.26	29	16.11	0.287	0.110	13.32	15.65	0.652	0.066
Heart failure	168	33.73	93	29.25	75	41.67	0.007	0.262	33.48	32.26	0.818	0.026
Atrial fibrillation	66	13.25	47	14.78	19	10.56	0.231	0.127	12.93	10.87	0.538	0.064
Peripheral artery disease	32	6.43	21	6.60	11	6.11	0.980	0.020	6.86	9.85	0.550	0.108
LVEF	57.17 ± 14.53		56.43 ± 14.01		58.48 ± 15.35		0.130	0.140	57.29 ± 17.89	58.12 ± 25.14	0.140	0.040
CAG finding							0.702	0.061			0.897	0.055
1-vessel disease	123	24.70	80	25.16	43	23.89	0.836	0.030	25.19	23.16	0.661	0.048
2-vessel disease	141	28.31	93	29.25	48	26.67	0.610	0.058	28.30	30.43	0.744	0.047
3-vessel disease	234	46.99	145	45.60	89	49.44	0.464	0.077	46.51	46.42	0.988	0.002
PCI procedure							0.258	0.115			0.644	0.061
Single lesion intervention	278	55.82	171	53.77	107	59.44			56.03	53.02		
Multiple lesions intervention	220	44.18	147	46.23	73	40.56			43.97	46.98		
Single-vessel PCI	331	66.47	209	65.72	122	67.78	0.713	0.044	67.80	66.08	0.771	0.036
Multi-vessel PCI	167	33.53	109	34.28	58	32.22			32.20	33.92		
Location of lesion treated												
LM	38	7.63	29	9.12	9	5.00	0.137	0.161	7.56	8.64	0.824	0.040
LAD	319	64.06	207	65.09	112	62.22	0.586	0.060	63.61	65.11	0.788	0.031
LCX	194	38.96	131	41.19	63	35.00	0.205	0.128	38.13	35.7	0.686	0.050
RCA	234	46.99	153	48.11	81	45.00	0.565	0.062	46.82	41.49	0.377	0.108
SVG	2	0.40	0	0.00	2	1.11	0.130	0.150	.	0.39	.	
Stent												
Bare metal stent	214	42.97	141	44.34	73	40.56	0.468	0.077	42.59	38.06	0.433	0.093
Everolimus-eluting stent	93	18.67	63	19.81	30	16.67	0.456	0.082	18.70	22.5	0.563	0.094
Zotarolimus-eluting stent	99	19.88	58	18.24	41	22.78	0.270	0.113	20.09	17.31	0.483	0.071
Biolimus-eluting stent	26	5.22	16	5.03	10	5.56	0.966	0.023	5.17	4.22	0.624	0.045
Siroliums-eluting stent	65	13.05	36	11.32	29	16.11	0.166	0.140	13.54	12.5	0.761	0.031
Medications												
Clopidogrel	271	54.42	175	55.03	96	53.33	0.786	0.034	54.05	59.41	0.369	0.108
Ticagrelor	227	45.58	143	44.97	84	46.67	0.786	0.034	45.95	40.59	0.369	0.108
Beta blocker	367	73.69	228	71.70	139	77.22	0.215	0.127	73.28	69.82	0.589	0.079
RAS inhibitor	283	56.83	170	53.46	113	62.78	0.055	0.190	56.15	54.57	0.807	0.032
Statin	405	81.33	246	77.36	159	88.33	0.004	0.294	81.26	73.38	0.297	0.189
PPI use	203	40.76	127	39.94	76	42.22	0.687	0.047	39.62	39.12	0.934	0.010

ASMD, absolute standardized mean difference; CABG, coronary artery bypass graft; CAG, coronary angiography; CKD, chronic kidney disease; ESRD, end stage renal disease; LAD, left anterior descending artery, LCX, left circumflex artery; LM, left main artery; LVEF, left ventricular ejection fraction; MI, myocardial infarction; PCI, percutaneous coronary intervention; PPI, proton pump inhibitor; RAS, renin angiotensin system; RCA, right coronary artery; STEMI, ST-segment elevation myocardial infarction. Everolimus-eluting stent: Xience, Promus/Synergy, Zotarolimus-eluting stent: Resolute integrity/Onyx, Biolimus-eluting stent: BioMatrix, Siroliums-eluting stent: Nobori/Ultimaster, Orsiro.

**Table 2:** Reasons for premature discontinuation of aspirin.

	All	≤ 1 month	> 1 month	*p* value
	N = 498 (%)	N = 318 (%)	N = 180 (%)	
Gastrointestinal bleeding	232 (46.59)	147 (46.23)	85 (47.22)	0.904
Other sites bleeding	35 (7.03)	17 (5.35)	18 (10.00)	0.077
Aspirin allergy or intolerance	53 (10.64)	42 (13.21)	11 (6.11)	0.021
Gastrointestinal upset or discomfort	48 (9.64)	18 (5.66)	30 (16.67)	<0.001
Need surgery or thrombocytopenia	13 (2.61)	8 (2.52)	5 (2.78)	1.000
Other or unknown causes	117 (23.49)	86 (27.04)	31 (17.22)	0.018

**Fig 1:**
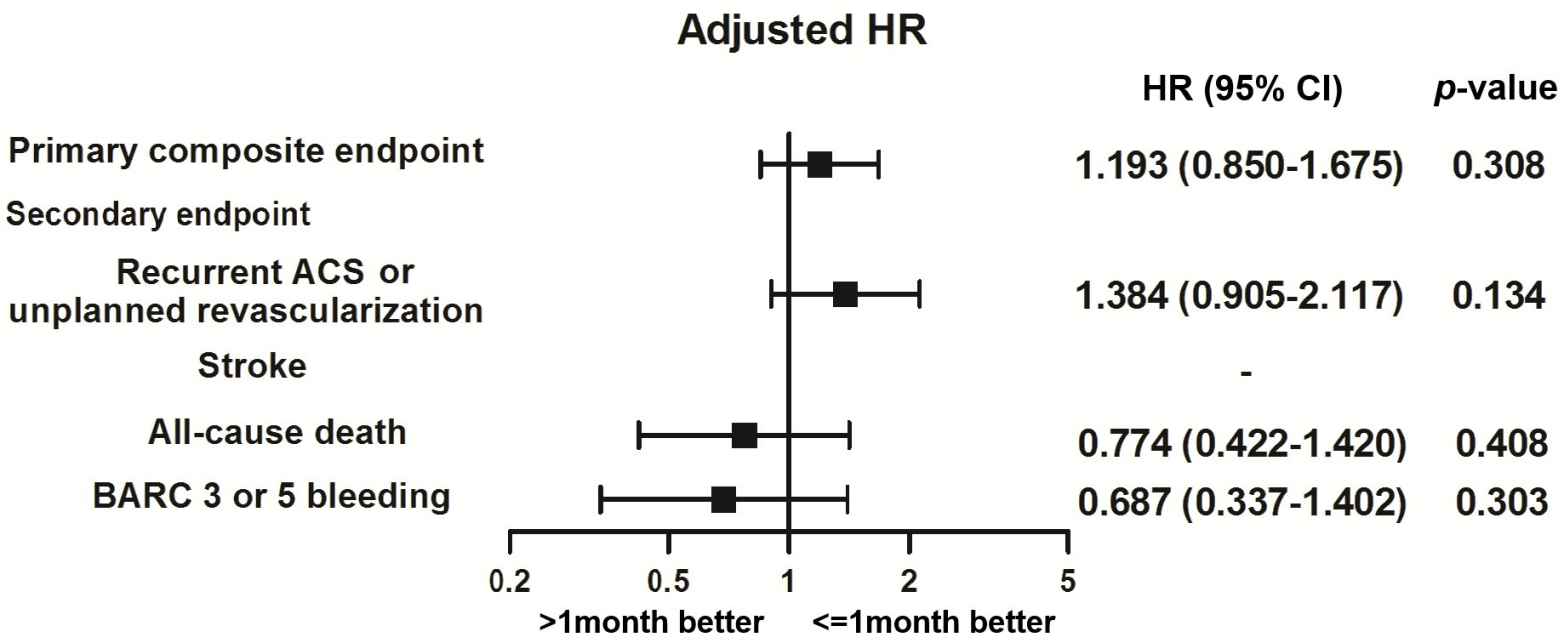
Clinical outcomes at 12-month follow up.

**Fig 2:**
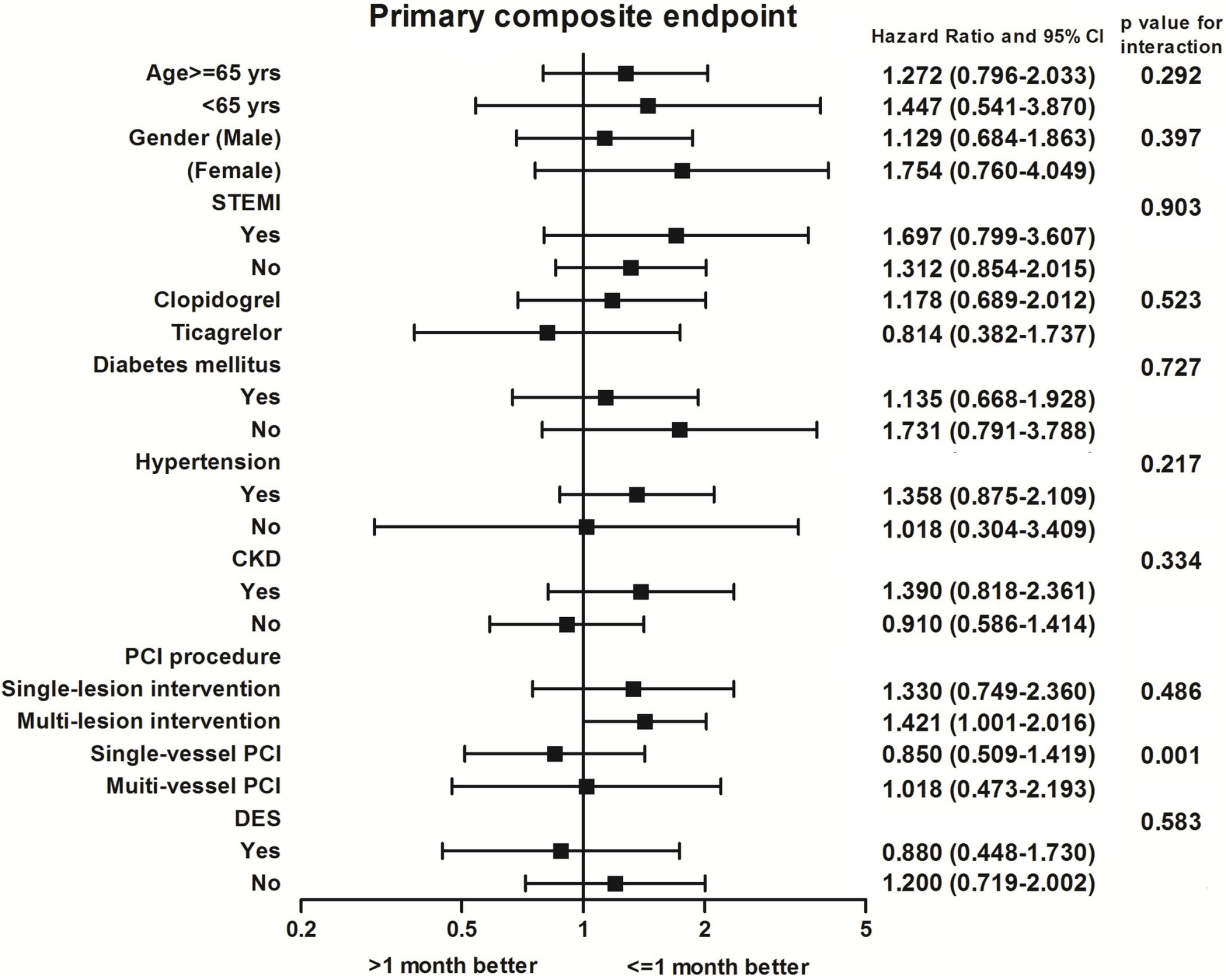
Subgroup analysis of the effect of different aspirin duration on primary composite endpoints.

The mean follow-up time was 336.75 ± 81.87 and 332.61 ± 75.75 days in each group (p = 0.578). The clinical outcomes during the 12-month follow-up were shown in **Table 3**. For primary composite endpoints, there were 40 events (12.6%) in those with aspirin treatment duration ≤ 1 month and 26 events (14.4%) in those with > 1 month. No significant difference was found between the groups after multivariate adjustment (adjusted HR 1.19, 95% CI 0.85–1.68). In the secondary endpoint, there were also no significant differences of recurrent ACS or unplanned revascularization and all-cause death between the groups. The risk of stroke was low with only 1 event. For safety outcome, there was one bleeding event of intracerebral hemorrhage and defined as BARC 5 bleeding. All other BARC 3 bleeding was gastrointestinal bleeding. Overall, the BARC 3 or 5 bleeding was 3.8% in those with aspirin treatment duration ≤ 1 month and 3.3% in those with > 1 month and there was no significant difference (adjusted HR 0.69, 95% CI 0.34–1.40) between the 2 groups. **Fig 1**shows the main findings of this study. Subgroup analysis showed that aspirin treatment duration ≤ 1 month had a consistent effect on the primary outcome across subgroups of age, sex, STEMI, clopidogrel or ticagrelor, diabetes mellitus, hypertension, chronic kidney disease, single or multiple-lesion intervention, and DES except in the subset of patients with multi-vessel PCI (Fig 2).

**Table 3:** Clinical outcomes at 12-month follow up.

	All	1≤ month	> 1 month	Crude HR	*p* value	Adjusted HR	*p* value
	N = 498	N = 318 (Ref)	N = 180	(95% CI)		(95% CI)	
Primary composite endpoint	66 (13.25)	40 (12.58)	26 (14.44)	1.304 (0.934–1.820)	0.119	1.193 (0.850–1.675)	0.308
Secondary endpoint							
Recurrent ACS or unplanned revascularization	41 (8.23)	24 (7.55)	17 (9.44)	1.625 (1.074–2.457)	0.022	1.384 (0.905–2.117)	0.134
Stroke	1 (0.20)	0	1 (0.56)	-		-	
All-cause death	24 (4.82)	16 (5.03)	8 (4.44)	0.722 (0.395–1.321)	0.291	0.774 (0.422–1.420)	0.408
BARC 3 or 5 bleeding	18 (3.61)	12 (3.77)	6 (3.33)	0.684 (0.337–1.387)	0.292	0.687 (0.337–1.402)	0.303

ACS, acute coronary syndrome; BARC, Bleeding Academic Research Consortium.

Adjusted variables included diabetes mellitus, previous PCI, peripheral artery disease, P2Y12 inhibitor, and statin.

## Discussion

This study analyzed the impact of different aspirin treatment duration on 12-month clinical outcomes in ACS patients received P2Y12 inhibitor monotherapy. Our results indicated that aspirin treatment > 1 month did not gain more ischemic risk reduction than those with aspirin treatment ≤ 1 month. The current recommendation of 12-month DAPT after ACS was mainly based on the previous clinical trials showing that, compared to aspirin monotherapy, DAPT reduced recurrent major adverse cardiovascular event (MACE) [17, 18]. The benefits of 12-month DAPT maybe no longer valid in the context of the recent progress in newer generation coronary stents, intracoronary imaging-guided optimal stent implantation and appearance of more potent P2Y12 inhibitors. Aspirin has been long considered to be the cornerstone of antiplatelet therapy. However, previous studies found aspirin added little additional inhibition of platelet aggregation under the treatment of potent P2Y12 inhibitors [19, 20]. Therefore, short-duration aspirin followed by P2Y12 inhibitor monotherapy become an alternative antiplatelet strategy in patients received PCI. The recent 5 clinical trials to test P2Y12 monotherapy strategy all designed to give aspirin in the first 1 or 3 months, traditionally regarded as the most vulnerable phase after PCI [7–11]. In our study, we found aspirin treatment ≤ 1 month (mean duration 7.52 ± 8.10 days) had similar ischemic outcomes to those having aspirin > 1 month (mean duration 98.05 ± 56.70 days). In patients with atrial fibrillation (AF) and PCI, recent meta-analysis studies indicated that omission of aspirin after PCI and use P2Y12 inhibitor plus oral anticoagulant not only reduced the risk of bleeding, but also carried no significant increase of MACE [21, 22]. In the post hoc analysis of the AUGUSTUS study for AF and PCI, use of aspirin up to 30 days resulted in more bleeding events but fewer ischemic events than placebo. However, prolonged use of aspirin over 30 days only increased bleeding risk, but without any significant benefit of reducing ischemic events [23]. Recently, a pioneer clinical trial in which patients with low-risk stable coronary artery disease were treated with prasugrel monotherapy without aspirin after elective PCI. No stent thrombosis was found after 3 months follow up in this study indicating aspirin-free strategy with P2Y12 inhibitor monotherapy may be feasible and safe in selected stable patients undergoing PCI [24]. Recent meta-analyses studies comparing P2Y12 inhibitor monotherapy vs. DAPT demonstrated that early aspirin discontinuation (1–3 months) with P2Y12 inhibitor monotherapy decreased bleeding risk and did not increase the risk of MACE, even in ACS patients [25, 26]. The remaining question is the optimal choice of P2Y12 inhibitor in patients prescribed monotherapy. It is well known that clopidogrel has a significant interpatient variability of antiplatelet activity [27]. In ACS patients under the background aspirin therapy, prasugrel and ticagrelor have better cardiovascular outcomes than clopidogrel [28, 29]. In ACS patients who received P2Y12 inhibitor monotherapy, our previous observation study showed that ticagrelor had a lower risk of ischemic outcome compared with clopidogrel during the 12-month follow up after PCI [13]. It seems that, if P2Y12 inhibitor monotherapy is adopted, a more potent P2Y12 inhibitor is a better choice than clopidogrel. Further randomized clinical trials are necessary to compare the efficacy and safety between ticagrelor vs. clopidogrel in P2Y12 inhibitor monotherapy.

One of the major limitations of our study is its non-randomized, observational study design and the study was not registered in a clinical trials database, such as ClinicalTrials.gov. Although the statistical method, IPTW, was used to balance the differences between the groups, some unmeasured or unidentified confounding factors still potentially may bias the clinical outcomes. For example, the duration of aspirin was not predefined in each group. The time to develop the reasons for stopping aspirin was variable. In about 23.5% patients, the true reasons for early aspirin discontinuation were unclear due to limited information recorded in the charts. Furthermore, only patients with available one-year follow-up data were included is another limitation because this cohort potentially could not represent the whole patient group with early aspirin interruption and selection bias could occur. Second, the small patient number is another major limitation of our study. It may cause a problem of underpower to evaluate the clinical events. Third, we found there was no significant difference in the risk of major bleeding (BARC type 3 to 5) between patients with longer or shorter aspirin treatment duration. In our study, all clinical events were investigator-reported, but not adjudicated by a clinical events committee. Recently, the Academic Research Consortium (ARC) for High Bleeding Risk (HBR) criteria were proposed and validated to identify patients with bleeding risk [30–32]. The rate of BARC 3 or 5 bleeding at 1 year was 3.6% in our cohort which can be defined as borderline HBR according to the 4% cut-off proposed by the ARC-HBR to identify HBR patients [31, 32]. Among the studies of P2Y12 inhibitor monotherapy, the SMART-CHOICE, STOPDAPT-2 and TICO trials were performed in East Asian countries. In the SMART-CHOICE study that compared aspirin plus a P2Y12 inhibitor for 3 months and followed by P2Y12 inhibitor monotherapy vs. DAPT for 12 months, the major bleeding risk defined as BARC type 3 to 5 bleeding was also similar between the groups (HR 0.87, 95% CI 0.40 to 1.88) [10]. In the STOPDAPT-2 compared 1-month DAPT followed by P2Y12 inhibitor monotherapy versus 12-month DAPT. The BARC type 3 or 5 bleeding was lower in the monotherapy group (HR 0.30, 95% CI 0.13–0.65), but the severe bleeding defined by GUSTO criteria was similar (HR 0.37, 95% CI 0.12–1.15) between the groups [9]. Only the TICO study exclusively included ACS patients [11]. The TICO study showed ACS patients received ticagrelor monotherapy after 3-month DAPT had a significantly lower risk of major bleeding (HR, 0.56, 95% CI 0.34–0.91). Overall, it is difficult to compare the results between these clinical trials and our study because of differences in P2Y12 inhibitor used and aspirin treatment duration. Fourth, 43% patients in this study received BMS and 54% patients still received clopidogrel. The data reflected current treatment status of ACS in Taiwan [33, 34]. The use of BMS is due to the restriction of the Taiwan National Health Insurance which only reimburses the price of BMS. Patients have to pay $1,500 to $2,000 US dollars for using one DES. For fear of bleeding, clopidogrel instead of ticagrelor, is still commonly used in most East Asian countries, including Taiwan. The ischemic outcome could be different if more ticagrelor and DES were used in our patients. Finally, we did not have the data about the percentage of patients that received complex PCI or complete revascularization. These factors cannot be analyzed in the subgroup analysis. There was a significant interaction in the subgroup analysis between single and multi-vessel PCI. Aspirin > 1 month was more favored in the subset of the patients with single vessel PCI. In the initial study protocol, we only recorded single lesion or multiple lesions intervention. The data of single or multi-vessel PCI was not recorded. We used the original CAG findings (1-vessel, 2-vessel, 3-vesssel disease), location of lesion treated, and PCI procedure (single or multiple lesions intervention) to roughly estimate the percentage of single or multivessel PCI. The influence of aspirin duration on clinical outcomes in these specific patient groups with different PCI procedures needs further investigation. We also did not know the effects of prasugrel monotherapy. Prasugrel was introduced into Taiwan in the end of 2018. There were only few patients received prasugrel during the study period, so no case of prasugrel monotherapy was included in this study.

## Conclusions

In conclusion, the risk of ischemic events was similar between those with aspirin treatment > 1 month versus ≤ 1 month in ACS patients undergoing PCI and received P2Y12 inhibitor monotherapy. Under P2Y12 inhibitor therapy, early discontinuation of aspirin ≤ 1 month after PCI may be feasible and safe. Due to the study’s limitations, further randomized clinical trials are needed to reconfirm our study results.
